# Beneficial effects of an angiotensin-II receptor blocker on structural atrial reverse-remodeling in a rat model of ischemic heart failure

**DOI:** 10.3892/etm.2013.920

**Published:** 2013-01-23

**Authors:** NAMSIK YOON, JEONG GWAN CHO, KYE HUN KIM, KEUN HO PARK, DOO SUN SIM, HYUN JU YOON, YOUNG JOON HONG, HYUNG WOOK PARK, JU HAN KIM, YOUNGKEUN AHN, MYUNG HO JEONG, JONG CHUN PARK

**Affiliations:** Department of Cardiovascular Medicine, The Heart Center of Chonnam National University Hospital, Chonnam National University Research Institute of Medical Science, Gwangju 501-757, Republic of Korea

**Keywords:** angiotensin-II receptor blocker, atrial fibrillation, atrial reverse-remodeling, heart failure

## Abstract

The remodeling of gap junctions may affect their conduction properties and contribute to the maintenance of atrial fibrillation. The significance of the role of angiotensin-II receptor blockers (ARBs) in upstream therapy is not clear. This study was performed to investigate the effects of ARBs on atrial remodeling in a heart failure model. A model of heart failure was established or sham surgery performed in 24 Sprague-Dawley male rats. The rats were divided into sham, heart failure and heart failure-ARB groups. In the ARB group, 30 mg/kg of losartan was administered each day for 4 weeks. Echocardiography was performed at the baseline and 4 weeks following the surgery. An atrial fibrillation induction study and histological and immunohistochemical evaluation were performed 4 weeks after surgery. The increase in the left atrial diameter of the heart failure-ARB group was smaller than that of the heart failure group (P=0.028). The atrial fibrillation inducibility and duration of induced atrial fibrillation were not different between the heart failure and heart failure-ARB groups. Masson’s trichrome staining revealed less fibrosis in the heart failure-ARB group compared with the heart failure group. Immunohistochemical staining and western blot analysis for connexin 43 showed a lower expression level in the heart failure-ARB group compared with that in the heart failure group. In a rat model of ischemic heart failure the ARB losartan had structural and histological atrial reverse-remodeling effects. However, its role as an electrical stabilizer requires further study.

## Introduction

Angiotensin-II receptor blockers (ARBs) are representative anti-hypertensive drugs. They are effective in left ventricular dysfunction and have reno-protective effects in the reduction of proteinuria and atrial reverse-remodeling effects as electrophysiological stabilizers (1). Studies concerning the reverse-remodeling effect are relatively rare and a number do not agree with each other in certain regards. Atrial fibrillation has a clear association with atrial remodeling. Angiotensin-converting enzyme lowers bradykinin levels, which increases fibrosis and collagen deposition in atrial tissue (2). Goette *et al* (3) demonstrated that the decrease in bradykinin levels is due to angiotensin-converting enzyme-dependent extracellular signal-regulated kinases (Erk1/Erk2). The activated angiotensin-II receptor activates mitogen-activated protein kinase. As a result of histological changes, atrial enlargement occurs and the atrium may be a substrate of atrial fibrillation (4). Electrical remodeling is another mechanism of atrial remodeling. Angiotensin activation induces myocyte calcium overload, which causes prolongation of the refractory period, depolarization-delay and an increase in automaticity. This also creates substrates for atrial fibrillation. In a similar manner, the renin-angiotensin-aldosterone system has a marked correlation with atrial remodeling which is closely associated with the development and maintenance of atrial fibrillation.

It is well known that hemodynamic overload in the atrium is one of the most important factors for atrial fibrosis (5) and structural changes, such as atrial enlargement and fibrosis, are associated with atrial dysfunction (6,7). Li *et al* (7) reported that electrical inhomogeneity due to atrial fibrosis had a significant role in atrial fibrillation induction and maintenance in a canine heart failure model. Gap junctions build signal propagation channels to neighboring myocytes. The geometrical distortion creates an inhomogeneous electrophysiological network and the subsequent consolidation of atrial fibrillation. These histological changes accompany atrial fibrosis and the expression of the proteins connexin 43 and connexin 40 (8). However, these results showed wide variations, with opposing results, even within the same models. Consequently, these studies were unable to define a definite causal relationship between arrhythmia and connexin (9).

Losartan has been reported to prevent left ventricular systolic dysfunction in a rat myocardial infarction model (10). The renin-angiotensin-aldosterone system is associated with the pathological mechanism of atrial fibrillation. Kumagai *et al* (1) reported that this ARB is able to prevent atrial electrical remodeling in canine models, in which rapid atrial pacing was used to induce atrial fibrillation.

The present study was performed to evaluate the reverse remodeling effect of an ARB by studying echocardiographic results, expression of cardiac connexin and atrial fibrillation inducibility in a heart failure model.

## Materials and methods

### Experimental animals and reagents

Male Sprague-Dawley rats (Jung-Ang Lab Animal Inc., Seoul, Korea) weighing ∼260 g were used in the experiments. Each rat was isolated and housed individually in a ventilated microisolator-cage rack system, in which day and night were set at 12 hourly intervals. The rats were fed with standard rodent provender and distilled water. The experiments were performed in accordance with the guidelines of the Chonnam National University Hospital Animal Subject Institutional Review Board (Gwangju, Korea). The amount of provender for each subject was estimated for 5 days of adaptation. The ground medicine was mixed into the ground provender, which was congealed for one day at 35°C.

A total of 38 rats were divided into sham (n=8), heart failure (n=15) and heart failure-angiotensin-II receptor blocker (ARB) (n=15) groups. Due to 14 rats not surviving surgery, each group ultimately contained 8 rats. Losartan potassium (30 mg/kg) was administered to the heart failure-ARB group for 4 weeks (10).

### Heart failure model

The ischemic heart failure model was induced using the method reported by Johns and Olson (11). Anesthesia was induced using an intramuscular injection of ketamine (50 mg/kg) and xylazine (5 mg/kg). Artificial ventilation was performed with a small animal mechanical ventilator (Harvard Apparatus, Holliston, MA, USA) following tracheal intubation. The rat heart was exposed after left thoracotomy in a supine position. Myocardial infarction was induced via ligation of the left anterior descending coronary artery in the heart failure and heart failure-ARB groups. The chest was closed immediately. In the sham group, only the thoracotomy and closure were performed. The rats were allowed to recover for 4 weeks.

### Echocardiography

**Figure 1:**
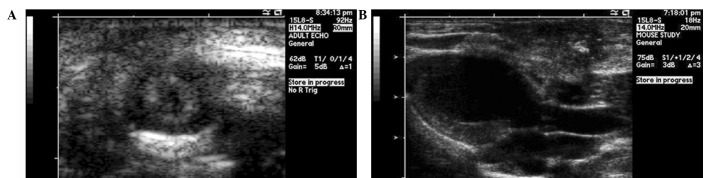
Echocardiography of (A) the parasternal short axis view and (B) the parasternal long axis view.

Echocardiography was performed under anesthesia using an intramuscular injection of ketamine (50 mg/kg) and xylazine (5 mg/kg) prior to surgery and 4 weeks after surgery. A 15 MHz linear probe (Sequoia C512; Acuson-Siemens, Mountain View, CA, USA) was used to perform transthoracic echocardiography. The left ventricular end systolic diameter (LVESd), left ventricular end diastolic diameter (LVEDd), left atrial diameter (LAD) in the parasternal long axis view and left ventricular ejection fraction (LVEF) were measured (Fig. 1A and B). All echocardiograpies were performed in blinded state.

### Induction of atrial fibrillation

**Figure 2:**
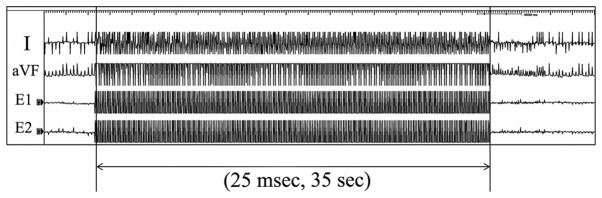
To induce atrial fibrillation, burst stimulation at a frequency of 25 msec interval was performed for 35 sec in each of the rats.

After follow-up echocardiography, atrial fibrillation induction was performed under anesthesia. Following a 10-min equilibration period after anesthesia, a 4F electrode catheter (St. Jude Medical, Inc., St. Paul, MN, USA) was inserted into the esophagus and positioned to ensure constant atrial capture. A BLOOM 215B DTU stimulator, Prucka recording system, Grass S-8800 2-channel stimulator (Grasstech, Quincy, MA, USA), MP150 and ECG100C ×2 (Biopac, Goleta, CA, USA) were used for stimulation and recording. Atrial pacing was performed at twice the diastolic threshold (10 V, 500 Ω, 20 mA), using 2 poles on the pacing catheter (pulse width, 5 msec). To induce atrial fibrillation, burst stimulation at a frequency of 25 msec intervals was performed for 35 sec in each of the rats (Fig. 2). The rats were allowed 5 min of recovery in the sinus rhythm between stimulations for respiratory and circulatory recovery. The induction test was performed 5 times in each rat. Atrial fibrillation inducibility and duration were measured.

### Histological analysis

Following the atrial fibrillation induction test, all rats were sacrificed. For 5 of the 8 rats in each group, the hearts were fixed using 10% formaldehyde perfusion into the abdominal aorta. Following formaldehyde perfusion, the hearts were excised immediately. The excised hearts were fixed in 10% formaldehyde. Hematoxylin and eosin (HE) and Masson’s trichrome staining, as well as immunohistochemical staining for connexin 43 (using rabbit polyclonal anti-C×43 antibody, 1/100; Zymed Laboratories, CliniSciences, Montrouge, France) were performed. Fibrosis and connexin expression were quantified using automatic computer graphics software (Image J^®^).

### Western blot analysis

For the western blot analysis of connexin 43, the two atria from 3 of the 8 rats in each group were excised following sacrifice via CO_2_ inspiration. Western blot analysis was performed on lyates from frozen tissue samples. NP buffer (1%) was used as the lysis buffer. Protein extracts (50 *μ*g) were used. Connexin 43 was detected using rabbit polyclonal anti-C×43 antibody (1/100, Zymed Laboratories).

### Statistical analysis

Statistical analysis was performed using the SPSS 15.0 software for Windows^®^. Mann-Whitney tests were used for comparisons between the groups. Intra-group analysis was performed using paired t-tests. P<0.05 was considered to indicate a statistically significant difference.

## Results

### Heart failure model

**Figure 3:**
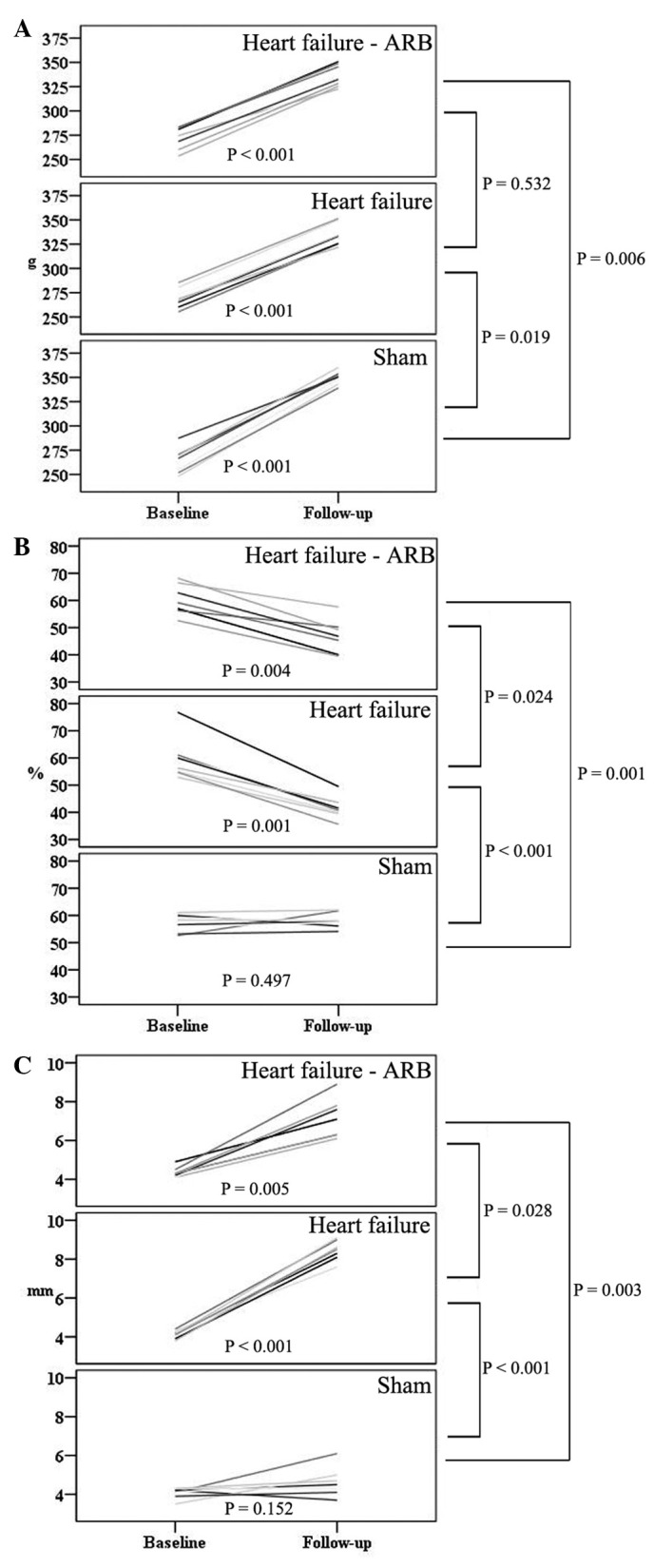
Serial changes in (A) body weight, (B) left ventricular ejection fraction and (C) left atrial diameter. ARB, angiotensin-II receptor blocker.

The rats were divided into sham (n=8), heart failure (n=15) and heart failure-ARB groups (n=15). After left anterior descending artery ligation, 14 rats died. A total of 8 rats were successfully maintained in each group for 4 weeks following surgery. There was no significant difference in weight gain between the heart failure and heart failure-ARB groups for all 4 weeks (Fig. 3A).

### Echocardiography

The baseline LVEFs in the sham, heart failure and heart failure-ARB groups were 56.6±2.78, 62.6±8.28 and 59.3±4.13%, respectively. The follow-up LVEFs were 58.2±2.08, 43.0±3.82 and 47.4±6.76%, respectively. The reduction in the LVEF of the heart failure-ARB group was significantly smaller than that in the heart failure group (P= 0.024; Fig. 3B). The baseline LADs in the sham, heart failure and heart failure-ARB groups were 4.0±0.29, 4.2±0.18 and 4.3±0.33 mm, respectively. The follow-up LADs were 4.8±0.87, 8.5±0.39 and 7.3±1.19 mm, respectively. The increase in the LAD of the heart failure-ARB group was significantly smaller than that in the heart failure group (P=0.028; Fig. 3C). The size of the left atrial appendage in the heart failure-ARB group was smaller than that in the heart failure group.

### Atrial fibrillation inducibility

**Figure 4:**
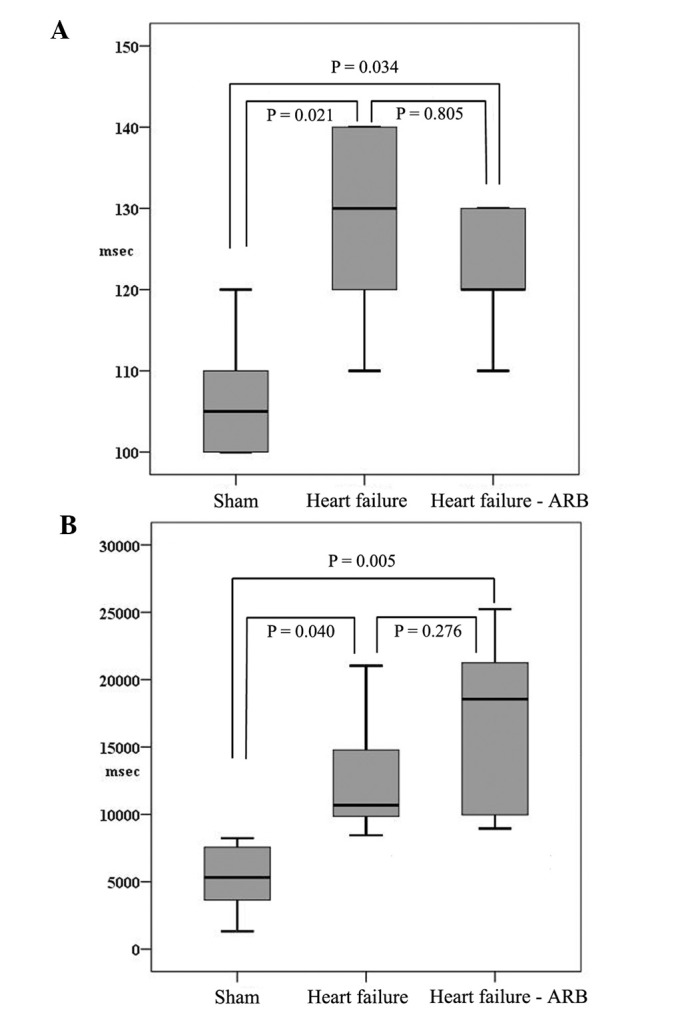
Comparison of (A) atrioventricular block cycle length and (B) duration of induced atrial fibrillation. ARB, angiotensin-II receptor blocker.

The atrioventricular block cycle lengths in the sham, heart failure and heart failure-ARB groups were 107±8.3, 124±11.4 and 116±11.4 msec, respectively (Fig. 4A). The inducibility of atrial fibrillation in the sham, heart failure and heart failure-ARB groups was 80±14, 91±11 and 92±10%, respectively. The durations of induced atrial fibrillation in the sham, heart failure and heart failure-ARB groups were 5213±2840.2, 12960±5088.8 and 16790±7112.8 msec, respectively (Fig. 4B).

### Histology, immunohistochemical staining and western blotting

**Figure 5:**
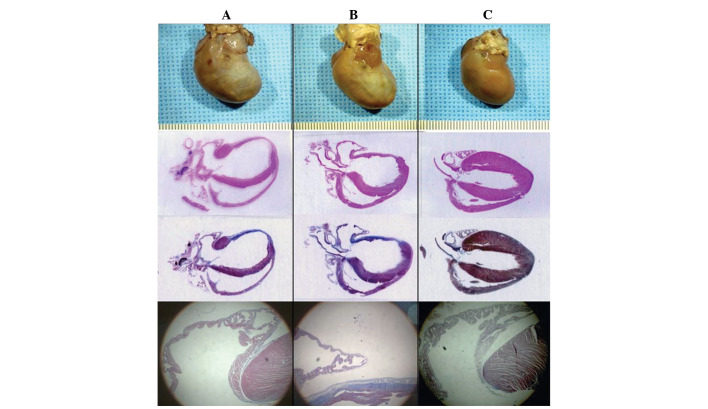
Photography after formalin fixation, gross view of HE staining, gross view of Masson’s trichrome staining and Masson’s trichrome staining of the left atrial appendage (from top to bottom, respectively; magnification, ×20). (A) Heart failure-ARB; (B) heart failure; and (C) sham groups. The gross sizes of the hearts in the heart failure groups were larger than those in the sham group. Whitish areas in the left ventricle were observed in the infarcted areas (A and B). Ventricular enlargement and wall thining in the infarcted areas were observed by HE staining (A and B). Fibrosis was observed by Masson’s trichrome staining (A and B). ARB, angiotensin-II receptor blocker; HE, hematoxylin and eosin.

The gross sizes of the excised hearts in the heart failure and heart failure-ARB groups were larger than those in the sham group. The area of infarction was pale and thin (Fig. 5).

**Figure 6:**
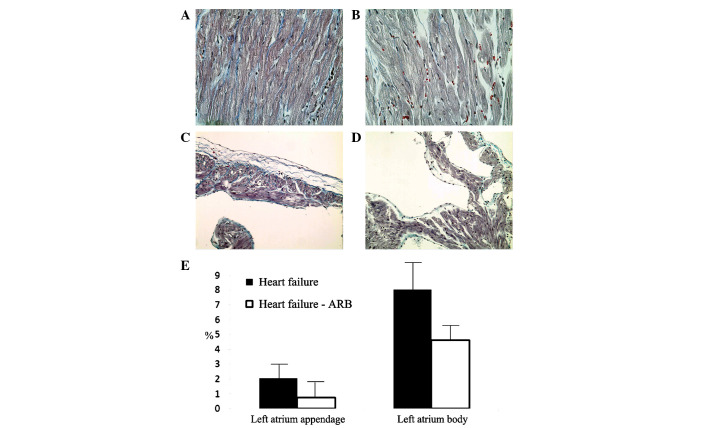
Representative Masson’s trichrome-stained sections of the left atrium from the (A and C) heart failure and (B and D) heart failure-ARB groups. In the heart failure group, extensive interstitial fibrosis was observed. Furthermore, the amount of connective tissue was increased and this extended around the parenchymal cells. In the heart failure-ARB group, interstitial fibrosis was attenuated. Magnification, ×400 (A and B) or ×200 (C and D). (E) Bar graph shows that the fibrosis in the heart failure-ARB group was less than that in the heart failure group. ARB, angiotensin-II receptor blocker.

In the HE staining (Fig. 5) of the heart failure group, significant dilatation of the atria and ventricles was evident, as was marked thinning of the wall of the infarcted area. The HE staining showed the destructive features of the myocardial contraction unit, abnormal sarcomeres and clear interstitial fibrosis. By contrast, the atrial myocytes were preserved relatively intact in the myocardial contraction unit with normal sarcomeres in the heart failure-ARB group (Fig. 6B and D).

The Masson’s trichrome staining revealed severe fibrosis of the infarcted ventricular area and more fibrosis of the atria in the heart failure group (Fig. 6A and B). The left atrial appendage areas of fibrosis in the heart failure and heart failure-ARB groups were 2.074 and 0.761%, respectively, according to Masson’s trichrome staining. The left atrial body areas of fibrosis in the heart failure and heart failure-ARB groups were 8.009 and 4.611%, respectively (Fig. 6E).

**Figure 7:**
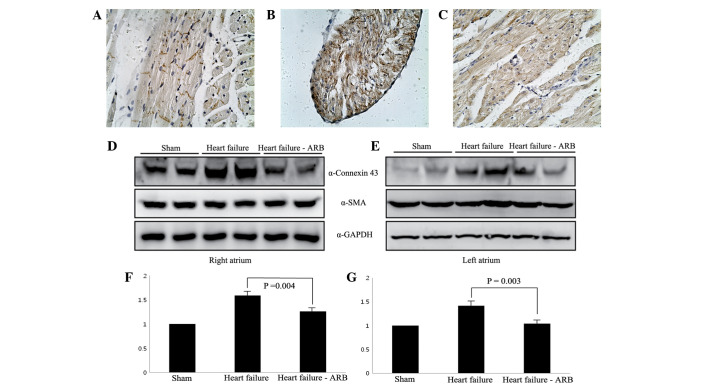
(A) Even distribution of connexin 43 protein was observed in the intercalated discs of the sham group (magnification, ×400). (B) Connexin 43 protein staining was not observed in the intercalated discs of the heart failure group (magnification, ×400). (C) A small amount of connexin 43 protein staining was observed in the intercalated discs of the heart failure-ARB group (magnification, ×400). (D and E) Western blot analysis of connexin 43 protein showed a lower level of expression in the heart failure-ARB group. (F and G) Bar graphs showed a significant difference in the expression of connexin 43. ARB, angiotensin-II receptor blocker.

The immunohistochemical staining revealed that connexin 43 protein was present in the intercalated discs in the sham group (Fig. 7A). Abundant plasmalemmal distribution of connexin 43 was observed in the heart failure group, while connexin 43 staining was not observed in the intercalated discs (Fig. 7B). In the heart failure-ARB group, a small amount of connexin 43 protein staining was observed in the intercalated discs (Fig. 7C).

Western blotting for connexin 43 showed that the connexin 43 bands of the heart failure group were more marked than those of the sham group and the heart failure-ARB group had weaker connexin 43 bands compared with the heart failure group (Fig. 7D–G).

## Discussion

Atrial fibrillation is highly associated with atrial remodeling. It is a relatively common arrhythmia, which may be paroxysmal and persistent. It develops with or without underlying heart diseases. Atrial fibrillation has clinical significance due to probable hemodynamic instabilities and thromboembolic events. The multiple-wavelet hypothesis has been the accepted theory for the mechanism of atrial fibrillation, whereby multiple wavelets conflict and collide, then create small wave-lets repeatedly (12).

Atrial fibrillation is induced by initial abnormal electrical activity and is maintained in the atrium with irregular reentry. These irregular electrical reentries are associated with structural and electrical remodeling. The ion channel-like protein connexin appears to be important in remodeling (13). This remodeling is frequent in myocardial infarction. The reason why atrial fibrillation develops frequently in old ventricular myocardial infarction is not fully understood (14). The current understanding is that the old ventricular myocardial infarction causes atrial structural and electrical remodeling, which act as substrates for atrial fibrillation. Old ventricular myocardial infarctions may induce electrical inhomogeneity and sympathetic over-distribution in the atrium without atrial infarction. These conditions appear to be sufficient to induce atrial fibrillation (14). With regard to this view, the rat ischemic heart failure model was induced by myocardial infarction via left coronary artery ligation in the present study. This was to create similar substrates to the theoretical conditions for atrial fibrillation maintenance. In humans, the dilatation of the infarcted ventricle develops alongside hypertrophy of the non-infarcted myocardium within months or years (15). In the present study, the thinning of the infarcted area and hypertrophy of the non-infarcted area developed in 28 days. The rapidity of these changes appears to be due to the high left ventricular systolic pressure, similar to that of humans, and the difference in myocardial thickness between humans and rats.

Atrial systolic function may not be recovered even when cardioversion is performed chemically or electrically. We suggest that the cause of this phenomenon is that structural and electrical remodeling of the atrium may not be reversed quickly. The fact that persistent atrial fibrillation induces chronic hibernation-like changes in the atrial myocardium may explain the slow reversal (6). Inhomogeneity of the gap junction is one of the structural remodelings in chronic atrial fibrillation. We propose that gap junction inhomogeneity is the result of atrial dilatation and fibrillation itself so that atrial fibrillation begets further atrial fibrillation. Consequently, atrial dilatation and fibrillation may be induced in ventricular failure. The remodeling of gap junctions may affect the conduction properties and subsequently contribute to the maintenance of atrial fibrillation. It is not clear how significant a role the remodeling has in the induction of atrial fibrillation (13). We hypothesize that gap junction remodeling is able to reduce conduction velocity in the atrium.

Thomas *et al* (16) reported that a 38% reduction in ventricular conduction velocity was observed when connexin 43 levels were reduced by 50%, indicating that connexin 43 has a significant role in electrical conduction between ventricular myocytes. By contrast, the electrical conduction velocity in the atrium was not affected by the reduction in connexin 43 levels but was affected by reductions in connexin 40 levels. Consequently, the authors suggested that connexin 43 is ventricle-specific and connexin 40 is atrium-specific. However, since their report, it has been recognized that connexin 40 and connexin 43 are associated with atrial fibrillation.

In the present study, connexin 43 exhibited an abundant plasmalemmal distribution in the heart failure group and a smaller connexin 43-stained area in the heart failure-ARB group. Additionally, western blotting showed that the connexin 43 bands of the heart failure group were more marked than those of the heart failure-ARB group. We hypothesize that the change in the quantity and distribution of connexin 43 is the result of changes in dephosphorylated connexin 43 levels in myocardial infarction or ischemic heart failure. Connexin 43 has multiple electrophoretic isoforms, including a non-phosphorylated and phosphorylated form. Phosphorylation regulates the assembly, degradation and gating of connexin 43 (17). During heart failure, large amounts of connexin 43 are hypophosphorylated and contribute to reducing the gap junction functions (18), causing electrical uncoupling, which promotes arrhythmogenicity. It appears that ARBs inhibit the dephosphorylation of connexin 43, thus reducing the levels of non-functional gap junctions in the rat heart failure model, and that this phosphorylation is important in gap junction coupling. The phosphorylation of connexin 43 is regulated by several protein kinases, including mitogen-activated protein kinase. Notably, the mechanism by which ARBs inhibit atrial fibrosis is due to the effect of this kinase. ARBs bind to the angiotensin-II receptor and inhibit mitogen-activated protein kinase; this inhibition, in turn, inhibits collagen accumulation, fibroblast proliferation and apoptosis (19). Also, the blocking of the angiotensin-II receptor inhibits electrical remodeling, by inhibiting calcium overload in the atrium.

Certain studies have reported the changes in connexin quantity or arrangement of various species (20) and the results have been extremely variable. The studies have reported conflicting results, even within the same models. We considered that this variability of results was due to the use of different species. Consequently, we reviewed several studies which used rat models. Hoyano *et al* (21) reported the downregulation of connexin 43 in an autoimmune myocarditis model while Reil *et al* (22) reported no change in connexin 43 levels in an aldosterone infusion model. Additionally, Rucker-Martin *et al* (23) reported the upregulation of connexin 43 in a heart failure model.

The results of the present study are compatible with the results of Rucker-Martin *et al*. Regardless of the connexin results, it is important for physicians to know whether ARBs have potential as an upstream therapy in atrial fibrillation. However, in the present induction study of atrial fibrillation, it was not possible to not identify a significant difference between heart failure and heart failure-ARB groups. Certain studies have reported that candesartan is able to prevent atrial fibrillation, and thus inhibit atrial structural remodeling (1). In a canine model study, the inhibition of the angiotensin-II receptor prevented the shortening of the atrial effective refractory period during rapid atrial pacing. This evidence demonstrates the association between angiotension II and atrial electrical characteristics (24). The 2010 European Society of Cardiology guidelines recommend that angiotensin converting enzyme inhibitors and angiotensin-II receptor blockers should be considered for the prevention of new-onset atrial fibrillation in patients with heart failure and reduced ejection fractions (25). The guidelines also suggest that there is now little reason to consider the use of upstream therapy for the prevention of atrial fibrillation recurrence in patients with little or no underlying heart disease (26).

The present study had several limitations. Losartan potassium is lipid-soluble, so if it is dissolved in water, an uneven distribution may be expected. However, ground medicine and provender were mixed for each rat individually. Also, whether the rats had eaten all their provender was checked daily.

Echocardiography is a sonographer-dependent technique. As such, there is a probability of inter-observer variation or open labeling. In the present study, all echocardiography was performed in a blinded state to attenuate such concerns.

The fact that an angiotensin converting enzyme inhibitor is able to inhibit left ventricular dilatation in patients with ventricular dysfunction following myocardial infarction has been well established by animal experiments and clinical trials (27,28). In the present experiment, the atrial response was evaluated in a left ventricular dysfunction model. Consequently, the atrial response may have been a secondary result from the improvement of left ventricle function. The prolonged inhibition of angiotensin converting enzyme and angiotensin-II receptor is able to reduce ventricular hypertrophy, recover coronary artery resistance and reduce ventricular fibrosis (29). Thus, in the present study, the angiotensin-II receptor blocking effect may have been the result of inhibition of not only atrial remodeling itself, but also left ventricular remodeling. In a heart failure model, it may be difficult to differentiate between these two effects. However, they may be differentiated in transgenic animal models, such as connexin knockouts or overexpression of Rho-A, Junctin, CRE modulator, angiotensin converting enzyme, constitutive TGF-b1 and Kir2.1. However, even in such models, there have been a number of contradictory results.

In conclusion, ARBs have a small preventive effect in atrial fibrillation if there is no underlying heart disease such as heart failure. However, ARBs also have an atrial reverse remodeling effect. It was revealed that, in a rat ischemic heart failure model, the ARB losartan had structural and histological atrial reverse-remodeling effects. It should be kept in mind that atrial fibrillation is a result of complex molecular, mechanical and electrical remodeling. Thus, we suggest that gap junctions may not be surrogate targets for atrial remodeling. The role of ARBs as electrical stabilizers requires further study.

## References

[1] Kumagai K, Nakashima H, Urata H, Gondo N, Arakawa K, Saku K (2003). Effects of angiotensin II type 1 receptor antagonist on electrical and structural remodeling in atrial fibrillation. J Am Coll Cardiol.

[2] Wollert KC, Drexler H (1997). The kallikrein-kinin system in post-myocardial infarction cardiac remodeling. Am J Cardiol.

[3] Goette A, Staack T, Röcken C (2000). Increased expression of extracellular signal-regulated kinase and angiotensin-converting enzyme in human atria during atrial fibrillation. J Am Coll Cardiol.

[4] Novo G, Guttilla D, Fazio G, Cooper D, Novo S (2008). The role of the renin-angiotensin system in atrial fibrillation and the therapeutic effects of ACE-Is and ARBS. Br J Clin Pharmacol.

[5] Boixel C, Fontaine V, Rücker-Martin C (2003). Fibrosis of the left atria during progression of heart failure is associated with increased matrix metalloproteinases in the rat. J Am Coll Cardiol.

[6] Ausma J, Wijffels M, Thoné F, Wouters L, Allessie M, Borgers M (1997). Structural changes of atrial myocardium due to sustained atrial fibrillation in the goat. Circulation.

[7] Li D, Fareh S, Leung TK, Nattel S (1999). Promotion of atrial fibrillation by heart failure in dogs: atrial remodeling of a different sort. Circulation.

[8] Dhein S (2006). Role of connexins in atrial fibrillation. Adv Cardiol.

[9] Gollob MH (2006). Cardiac connexins as candidate genes for idiopathic atrial fibrillation. Curr Opin Cardiol.

[10] Daniëls MC, Keller RS, de Tombe PP (2001). Losartan prevents contractile dysfunction in rat myocardium after left ventricular myocardial infarction. Am J Physiol Heart Circ Physiol.

[11] Johns TN, Olson BJ (1954). Experimental myocardial infarction. I. A method of coronary occlusion in small animals. Ann Surg.

[12] Jalife J (2003). Experimental and clinical AF mechanisms: bridging the divide. J Interv Card Electrophysiol.

[13] Takeuchi S, Akita T, Takagishi Y (2006). Disorganization of gap junction distribution in dilated atria of patients with chronic atrial fibrillation. Circ J.

[14] Miyauchi Y, Zhou SM, Okuyama Y (2003). Altered atrial electrical restitution and heterogeneous sympathetic hyperinnervation in hearts with chronic left ventricular myocardial infarction: implications for atrial fibrillation. Circulation.

[15] Mitchell GF, Lamas GA, Vaughan DE, Pfeffer MA (1992). Left ventricular remodeling in the year after first anterior myocardial infarction: a quantitative analysis of contractile segment lengths and ventricular shape. J Am Coll Cardiol.

[16] Thomas SA, Schuessler RB, Berul CI (1998). Disparate effects of deficient expression of connexin43 on atrial and ventricular conduction: evidence for chamber-specific molecular determinants of conduction. Circulation.

[17] Solan JL, Lampe PD (2005). Connexin phosphorylation as a regulatory event linked to gap junction channel assembly. Biochim Biophys Acta.

[18] Ai X, Pogwizd SM (2005). Connexin 43 downregulation and dephosphorylation in nonischemic heart failure is associated with enhanced colocalized protein phosphatase type 2A. Circ Res.

[19] Savelieva I, Camm AJ (2004). Atrial fibrillation and heart failure: natural history and pharmacological treatment. Europace.

[20] Kato T, Iwasaki YK, Nattel S (2012). Connexins and atrial fibrillation: filling in the gaps. Circulation.

[21] Hoyano M, Ito M, Kimura S (2010). Inducibility of atrial fibrillation depends not on inflammation but on atrial structural remodeling in rat experimental autoimmune myocarditis. Cardiovasc Pathol.

[22] Reil JC, Hohl M, Selejan S (2012). Aldosterone promotes atrial fibrillation. Eur Heart J.

[23] Rucker-Martin C, Milliez P, Tan S (2006). Chronic hemodynamic overload of the atria is an important factor for gap junction remodeling in human and rat hearts. Cardiovasc Res.

[24] Nakashima H, Kumagai K, Urata H, Gondo N, Ideishi M, Arakawa K (2000). Angiotensin II antagonist prevents electrical remodeling in atrial fibrillation. Circulation.

[25] Camm AJ, Kirchhof P, Lip GYH, European Heart Rhythm Association; European Association for Cardio-Thoracic Surgery (2010). Guidelines for the management of atrial fibrillation The Task Force for the Management of Atrial Fibrillation of the European Society of Cardiology (ESC). Europace.

[26] Camm AJ, Lip GY, De Caterina R (2012). 2012 focused update of the ESC Guidelines for the management of atrial fibrillation: an update of the 2010 ESC Guidelines for the management of atrial fibrillation - developed with the special contribution of the European Heart Rhythm Association. Europace.

[27] Nicolosi GL, Latini R, Marino P (1996). The prognostic value of predischarge quantitative two-dimensional echocardiographic measurements and the effects of early lisinopril treatment on left ventricular structure and function after acute myocardial infarction in the GISSI-3 trial. Gruppo Italiano per lo Studio della Sopravvivenza nell’Infarto Miocardico. Eur Heart J.

[28] St John Sutton M, Pfeffer MA, Plappert T (1994). Quantitative two-dimensional echocardiographic measurements are major predictors of adverse cardiovascular events after acute myocardial infarction. The protective effects of captopril. Circulation.

[29] Schieffer B, Wirger A, Meybrunn M (1994). Comparative effects of chronic angiotensin-converting enzyme inhibition and angiotensin II type 1 receptor blockade on cardiac remodeling after myocardial infarction in the rat. Circulation.

